# Emergence Angle, Marginal Bone Loss, and Radiographic Corticalization Around MIS Implants: A 5-Year Retrospective Study of Single, Splinted, and Bridge Restorations †

**DOI:** 10.3390/jcm15103764

**Published:** 2026-05-14

**Authors:** Tomasz Wach, Klaudia Kuta, Adam Michcik, Piotr Hadrowicz, Piotr Szymor, Paulina Pruszyńska, Grzegorz Trybek, Maciej Sikora, Marcin Kozakiewicz

**Affiliations:** 1 Department of Maxillofacial Surgery, Medical University of Lodz, 251 Pomorska Str., 92-213 Lodz, Poland; piotr.szymor@umed.lodz.pl (P.S.); paulina.pruszynska@umed.lodz.pl (P.P.); marcin.kozakiewicz@umed.lodz.pl (M.K.); 2 Dental Technology Program, Faculty of Medicine, Medical University of Lodz, Poland, 251 Pomorska Str., 92-213 Lodz, Poland; klaudia.kuta1@student.umed.lodz.pl; 3 Department of Maxillofacial Surgery, Medical University of Gdansk, 80-210 Gdańsk, Poland; adammichcik@gumed.edu.pl; 4 Department of Otolaryngology, Hospital in Sosnowiec, Zegadłowicza 3, 41-200 Sosnowiec, Poland; phadrowicz@gmail.com; 5 Department of Oral Surgery, Pomeranian Medical University in Szczecin, 70-111 Szczecin, Poland; g.trybek@gmail.com; 6 Department of Maxillofacial Surgery, 4th Military Clinical Hospital in Wroclaw, ul. Rudolfa Weigla 5, 50-981 Wroclaw, Poland; 7 National Medical Institute of the Ministry of Interior and Administration, Wołoska 137 Str., 02-507 Warsaw, Poland; sikora-maciej@wp.pl; 8 Department of Maxillofacial Surgery, Hospital of the Ministry of Interior, Wojska Polskiego 51, 25-375 Kielce, Poland; 9 Department of Biochemistry and Medical Chemistry, Pomeranian Medical University, Powstańców Wielkopolskich 72, 70-111 Szczecin, Poland

**Keywords:** texture analyses, dental implants, crown emergence angle, marginal bone loss, corticalization index, prosthetic restoration, single crowns, splinted crowns, bridges

## Abstract

**Background/Objectives**: Dental implants are a predictable treatment modality for missing teeth, and the crown emergence angle may affect peri-implant tissue health. This study evaluated the influence of crown emergence angle on marginal bone loss (MBL) and the corticalization index (CI) at 3-month and 5-year follow-ups, considering restoration type and implant location. **Methods**: Records from 155 patients treated with MIS implants were analyzed retrospectively. The primary outcome was marginal bone loss (MBL) at 60 months after loading; secondary/exploratory outcomes included MBL at 3 months and the Corticalization Index (CI) at 3 and 60 months. Three prosthodontic restorations were included: single crowns, splinted crowns, and bridges. Intraoral radiographs were used to measure crown emergence angle, MBL, and CI. **Results**: Recorded emergence profile was 32° ± 10°. Marginal bone loss increased over time but did not differ significantly among restoration groups. No significant relationship was found between emergence angle and MBL for single crowns (*p* = 0.369) or splinted crowns (*p* = 0.176). For bridges, a weak but statistically significant relationship was observed (*p* = 0.042). CI increased over time in all groups, without significant differences by restoration type. Restoration type was significantly associated with anterior/posterior implant location (*p* = 0.0004): single crowns predominated anteriorly, whereas splinted crowns and bridges were more frequent posteriorly. No significant association was found for maxilla versus mandible (*p* = 0.077). **Conclusions**: Within the limitations of this retrospective radiographic analysis, no clinically meaningful association was observed between emergence angle and MBL for single or splinted crowns. For bridges, the observed association was statistically significant but weak and should be interpreted cautiously. Other biological, biomechanical, and systemic factors may play a greater role in peri-implant tissue stability than emergence angle alone.

## 1. Introduction

Dental implants have significantly expanded the treatment options for patients who are fully or partially edentulous. They have enabled new therapeutic approaches, particularly in cases that were previously difficult to manage or where available treatment methods were insufficient. The predictability and long-term effectiveness of dental implants have been well documented for both fixed and removable prosthetic restorations. Most studies report that dental implants placed in fully or partially edentulous patients achieve success rates exceeding 90% [1].

A fundamental factor for treatment success in prosthodontics and implantology is the process of osseointegration. This refers to the biological bonding of the implant with the jawbone, mediated by osteogenic cells. Achieving osseointegration depends on two sequential stages of stabilization: primary and secondary. Primary (mechanical) stabilization is the initial state, achieved immediately during implant placement by tightly seating the implant in the prepared bone bed. Its effectiveness is influenced by the implant’s shape, thread design, and the bone density [1,2,3].

Secondary stabilization, in contrast, is a dynamic process that replaces mechanical primary stabilization with biological stability. While primary stabilization is purely mechanical, secondary stabilization develops over the healing period as new bone forms directly on the implant surface (contact osteogenesis) and undergoes remodeling. This process, driven by the biological response to the implanted foreign body, gradually enhances the actual structural integration between the bone and the implant. As a result, mechanical forces are transmitted through a direct microscopic connection rather than solely through friction [2,4].

Bone is a tissue that responds to various factors, including systemic changes in the body and local mechanical forces. Bone remodelling following implant placement is a process of resorption and formation occurring in the same location, replacing pre-existing bone. This remodelling process is responsible for improving bone quality around the implant. Both bone remodelling and modelling are primarily regulated by mechanical forces transmitted through implant loading [2,5].

According to Frost’s fundamental mechanostat theory, bone adapts its mass and architecture in response to mechanical stresses, maintaining homeostasis. Studies have shown that in areas where microdamage occurs (e.g., due to fatigue of the bone or material), the remodelling process is accelerated. Microdamage triggers biological signals that enhance bone turnover, resembling the healing processes of other tissues following injury [5].

The rate of bone remodelling, or Bone Remodeling Rate (BRR), depends on the type of bone and its response to loading. Woven bone forms more rapidly than lamellar bone. Higher BRR occurs in regions of mild overload, known as adaptive zones, where bone forms in response to mechanical stresses. These conditions promote the formation of more organized and mineralized bone, thereby enhancing implant stability. If an implant is properly designed so that the loads generate micro deformations within this adaptive zone, the bone at the implant interface will remain strong and well-organized, ensuring stable implant fixation under functional loading [2,5].

One such process occurring around implants subjected to occlusal loading is corticalization. This is an important adaptive process involving an increase in the density and mineralization of marginal bone around the implant under functional load. As a manifestation of bone remodeling in response to mechanical forces, corticalization leads to the development of a structure more closely resembling cortical bone. The Corticalization Index (CI) is used to quantitatively assess this phenomenon based on radiographic texture analysis [6,7,8].

In this study, implants from Medical Implants Technologies (MIS) were analyzed. These implants feature a tapered or cylindrical design, a double thread to enhance retention, and a rough, sandblasted surface. They are made from a titanium alloy, while the Premium version is composed of pure titanium, minimizing the risk of allergic reactions. The product line includes various models, including the C1 for standard implantation, MIS 7 with rings supporting soft tissue, M4 BioCom for soft bone, the one-piece Uno for narrow spaces, as well as temporary and orthodontic implants [9].

In prosthodontics, the concepts of emergence profile and crown emergence angle are particularly important. The emergence profile refers to the area where the implant transitions through the mucosa and is critical for soft tissue support and aesthetics. In implant prosthodontics, the emergence profile is a broader restorative concept, whereas the emergence angle (EA) represents a measurable geometric parameter of that profile. In the present study, the radiographic analysis focused specifically on the emergence angle. A distinction is made between the biological contour (concave, at the abutment) and the aesthetic contour (convex, toward the gingival margin). Natural teeth typically have a straight profile in the cervical region and an emergence angle of approximately 15° [2,4]. Proper shaping of this zone is essential for tissue health, aesthetics, and ease of hygiene. Previous studies suggest that a convex profile and an emergence angle exceeding 30° may increase the risk of soft tissue inflammation and marginal bone loss [10,11]. Traditionally, the profile is assessed using two-dimensional radiographs, which allow precise measurement of the mesial and distal emergence angles of the prosthetic restoration. This measurement method was applied in the present study.

Although the relationship between prosthetic emergence angle and marginal bone loss has been addressed in previous retrospective studies, most available reports have focused primarily on linear bone-level changes and have not simultaneously assessed radiographic bone-texture adaptation over long-term follow-up. Therefore, the present study was designed to evaluate emergence angle in relation not only to marginal bone loss (MBL), but also to the Corticalization Index (CI), in order to provide a broader radiographic perspective on peri-implant bone adaptation according to prosthetic restoration type.

The primary outcome of this retrospective study was marginal bone loss (MBL) at 60 months after prosthetic loading. Secondary and exploratory outcomes included MBL at 3 months and the Corticalization Index (CI) at 3 and 60 months, allowing a broader radiographic assessment of peri-implant bone adaptation over time. A novel aspect of this research is the combination of the conventional MBL parameter with the Corticalization Index assessment, enabling a more comprehensive evaluation of bone tissue adaptation to functional loading depending on the prosthetic solution employed.

## 2. Materials and Methods

The crown emergence angle is a key parameter in evaluating the aesthetics and functionality of prosthetic restorations on implants. Its value influences the stability of soft tissues, the hygiene of the peri-gingival region, and the risk of peri-implantitis. Accurate measurement of this angle allows assessment of restoration quality and its impact on the health of tissues surrounding the implant [10].

Because the study was based on anonymized retrospective radiographic documentation, no individual informed consent was required under the applicable local regulatory and ethical framework.

The aim of the study was to assess the influence of the prosthetic crown emergence angle on selected peri-implant bone parameters.

The analysis included documentation from 155 patients who underwent implant placement with delayed prosthetic restoration, categorized into three groups:

Group I: Patients with single crowns on implants.

Group II: Patients with splinted crowns on implants.

Group III: Patients with implant-supported fixed partial dentures (bridges).

The observation period for patients ranged from 3 months to 5 years following the loading of the prosthetic restoration. The 3-month follow-up was selected as an early post-loading radiographic time point to capture initial peri-implant bone adaptation, whereas the 60-month follow-up represented the principal long-term endpoint. These two time points were chosen to reflect early and long-term radiographic bone-related changes under functional loading. Inclusion in the study required complete radiographic documentation (intraoral X-rays) from the follow-up period. Exclusion criteria included unclear or insufficient-quality radiographs that prevented precise measurements, as well as factors known to significantly affect bone formation, remodelling, and implant biomechanics, in line with assumptions adopted in similar bone texture analyses [7,8]. These factors included systemic diseases substantially altering bone metabolism (e.g., diabetes, osteoporosis), previous radiotherapy in the head and neck region, active bisphosphonate therapy, diagnosed bruxism. Excluding such cases was intended to minimize the influence of confounding variables on the assessment of the relationship between emergence angle and peri-implant bone parameters [8]. Diagnosed bruxism was defined as a prior clinical diagnosis documented in the patient’s medical record; no additional instrumental assessment was performed for the purpose of this retrospective study.

To reduce confounding, the analysis was restricted to a single implant system (MIS) and excluded conditions known to substantially affect bone metabolism or implant biomechanics. Additional variables available in the retrospective dataset for descriptive characterization included implant diameter, implant length, insertion torque, implant location, and smoking status. However, some potentially relevant prosthetic and clinical covariates, including abutment-related and platform-related characteristics, retention mode, and oral hygiene indices, were not consistently available in the retrospective records and could therefore not be incorporated into the present analysis. Because of the retrospective nature of the study, incomplete availability of some covariates across observations and follow-up time points, and the exploratory analytical framework, these variables were not incorporated into a formal multivariable model. Therefore, the results should not be interpreted as evidence of a fully adjusted independent effect of emergence angle. Smoking status was extracted from the available retrospective records and categorized descriptively as smoker or non-smoker. No quantitative stratification according to smoking intensity or pack-years was consistently available in the dataset. Clinical evidence indicates that abutment design may influence post-prosthetic bone resorption and periodontal parameters; however, abutment-related variables were not consistently available in the present retrospective dataset and were therefore not included in the formal analyses [12].

Intraoral radiographs were obtained following a strict, standardized protocol and were performed by a single operator. The DIGORA OPTIME system (SOREDEX, Helsinki, Finland) DXR-50 model was used for all X-ray examinations. Exposure parameters were identical for all images: 7 mA, 70 kV, with an exposure time of 0.1 s. The X-ray device used in the study was manufactured by Instrumentarium Dental (Tuusula, Finland). To ensure reproducibility of results and comparability of images across different follow-up periods, specialized positioning systems were employed. These systems ensured that the X-ray beam was directed at a perpendicular angle (90°) to the phosphor plate for each measurement. This approach minimized projection errors and enabled reliable assessment of changes occurring in the alveolar bone surrounding the implants [9]. However, this radiographic method allowed assessment only of the mesial and distal interproximal aspects visible on intraoral radiographs and did not permit evaluation of the buccal or lingual/palatal bone plates.

Accordingly, the standardized radiographic protocol was intended to minimize projection-related variability in longitudinal mesial/distal measurements rather than to provide a three-dimensional assessment of peri-implant tissues.

All images were imported into the free software ImageJ (National Institutes of Health, Bethesda, MD, USA), version 1.54g. Image processing and region-based radiographic assessment were performed in the same standardized digital environment, whereas calculation and interpretation of the Corticalization Index followed the previously published texture-analysis framework described by Kozakiewicz et al. [7] and subsequently applied in implant-related radiographic studies [8].

**Figure 1:**
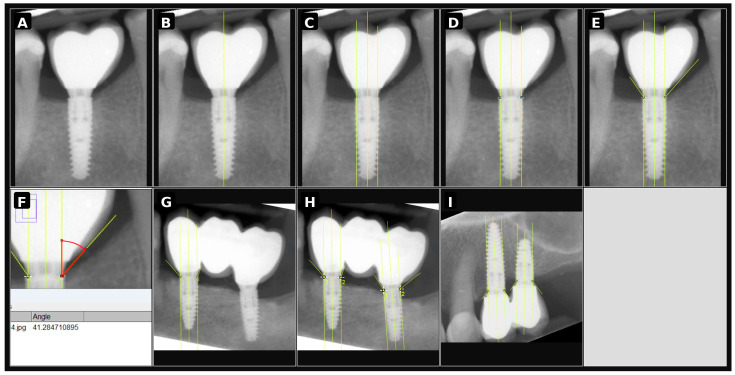
Step-by-step radiographic procedure for measuring the crown emergence angle. (**A**) Alignment of the image along the implant long axis; (**B**) identification of the implant long axis; (**C**) determination of the reference lines parallel to the implant axis; (**D**) identification of the implant-abutment junction; (**E**) drawing of the tangent line to the crown contour; (**F**) measurement of the angle between the implant axis and the crown tangent; (**G**,**H**) example of measurement in a bridge restoration; (**I**) example of measurement in splinted crowns.

The procedure for measuring the crown emergence angle for each implant was carried out according to the following protocol:

1. Image alignment along the implant long axis: The image was rotated so that the implant’s long axis was vertical (Figure 1A).
2. Identification of the implant long axis: A vertical line was drawn through the geometric center of the implant, extending from the apex to the center of the abutment platform (Figure 1B).
3. Reference lines determination: Auxiliary lines were drawn parallel to the implant long axis on the mesial and distal sides (Figure 1C).
4. Identification of the contact point: The implant-abutment junction (implant platform) was located on both the mesial and distal sides (Figure 1D).
5. Drawing tangent lines: A tangent line was drawn through each contact point, following the contour of the clinical prosthetic crown (Figure 1E).
6. Angle measurement: Mesial and distal emergence angle measurements were obtained separately, and the arithmetic mean of these two values was used as the final emergence angle for the respective implant/restoration unit in the statistical analyses. The angle in degrees was recorded using the Analyze > Measure option (Figure 1F).
7. For prosthetic bridges: The crown emergence angle was measured for each abutment of the bridge. An example measurement is shown in Figure 1G,H.
8. For splinted crowns: The measurement was performed similarly, taking into account the specific characteristics of the crown connections. An example is shown in Figure 1I.

For each implant, in addition to the emergence angle, the following parameters were assessed:

1. Marginal Bone Loss (MBL) was measured separately on the mesial and distal aspects as the vertical distance between the implant platform and the deepest point of bone loss. For analytical purposes, the arithmetic mean of the mesial and distal measurements was used as the final MBL value for the respective implant/restoration unit.
2. Corticalization Index (CI): a quantitative radiographic texture-based parameter describing changes in the architecture and density of the marginal peri-implant bone. CI was derived from standardized intraoral radiographs using a previously described and validated texture-analysis methodology applied to the peri-implant crestal bone region [7,8].

Radiographic measurements were performed using a standardized protocol and predefined measurement criteria. Emergence angle measurements were performed by one examiner, whereas marginal bone loss (MBL) and Corticalization Index (CI) measurements were performed by another examiner. Repeated measurements demonstrated approximately 90% repeatability. No formal inter-observer reliability analysis using agreement coefficients was performed.

The main outcome of interest was marginal bone loss (MBL) at 60 months, selected as the principal long-term radiographic endpoint. Secondary/exploratory outcomes included MBL at 3 months and the Corticalization Index (CI) at 3 and 60 months. Because of the retrospective design, no formal a priori sample size calculation was performed. The study sample therefore consisted of all eligible cases with complete radiographic documentation available for the predefined follow-up period. The statistical analyses were planned retrospectively on the basis of the available dataset and should be interpreted as exploratory rather than confirmatory.

Because emergence angle and marginal bone-related measurements were initially recorded separately on the mesial and distal aspects, the raw radiographic dataset contained interproximal side-specific measurements. However, for the principal statistical analyses, the arithmetic mean of the mesial and distal values was used as the final value for the respective implant/restoration unit. Consequently, the number of observations varied across analyses depending on the endpoint, follow-up time point, and availability of complete data. The present statistical approach did not include mixed-effects modelling or generalized estimating equations to adjust for intra-patient clustering; therefore, the inferential analyses should be interpreted as exploratory.

Statistical analyses were performed using STATISTICA software, version 13.1 (TIBCO Software Inc., Palo Alto, CA, USA). Descriptive statistics were calculated for all study variables. Group comparisons were performed using analysis of variance or the Kruskal–Wallis test, as appropriate for the distribution and structure of the analyzed data, and simple regression models were used to explore the association between emergence angle and long-term MBL within each prosthetic restoration group. A two-sided significance level of *p* < 0.05 was adopted. Given the retrospective nature of the study, the analyses should be interpreted as exploratory. Because the distribution of MBL values was markedly skewed, with a predominance of low or zero values and substantial inter-observation variability, exploratory regression analyses were performed using transformation-based functional forms available in the statistical software to describe potential non-linear associations between emergence angle and MBL at 60 months within each prosthetic subgroup. These models were used for descriptive/exploratory purposes and were not intended as confirmatory predictive models.

## 3. Results

A total of 536 mesial and distal emergence-angle measurements were available in the raw radiographic dataset. For the principal statistical analyses, mesial and distal values were averaged to obtain a single final value for each implant/restoration unit. Therefore, the denominator varied across tables and models depending on the endpoint analyzed, the follow-up time point, and the availability of complete data. Statistical analyses were performed using STATISTICA version 13.1, with a significance level set at *p* < 0.05.

Table 1 presents the basic descriptive statistics for the studied variables. The mean emergence angle was 31.8 ± 10.4° (median: 31.3°), ranging from 6.2° to 79.1°.

For marginal bone loss (MBL), a gradual increase over time was observed. At baseline (00M), the mean MBL was 0.0 ± 0.0 mm (median: 0 mm), at 3 months (03M) 0.2 ± 0.7 mm (median: 0 mm), and at 60 months (60M) 0.9 ± 1.2 mm (median: 0.430 mm). The large standard deviations, particularly for MBL at 03M (0.7 mm) and 60M (1.2 mm), along with the wide range of values (0.000 to 8.000 mm at 03M), indicate substantial heterogeneity in patient responses. Most patients exhibited minimal bone loss, while a few cases showed extremely high values.

The Corticalization Index (CI) increased systematically over time (Table 1). At 3 months (03M), the mean CI was 201 ± 126 (median: 165), while at 60 months (60M) it reached 295 ± 180 (median: 234). Despite the observed increase in mean and median values, the large standard deviations indicate substantial inter-individual variability among patients.

The characteristics of the implants used are detailed in Table 2. The most frequently used implant was the MIS 7 model (173 units) with a length of 10 mm and a diameter of 3.75 mm, followed by the MIS C1 implant (115 units) with a length of 13 mm and a diameter of 3.75 mm. The mean insertion torque was 40 ± 11 Ncm (median: 45 Ncm), with a range from 10 to 75 Ncm. The wide range reflects variations mainly in bone density.

The reported counts reflect the number of observations for which complete data on the respective implant-related variable were available in the retrospective records.

Among the available implant-related variables, insertion torque did not differ significantly among the prosthetic restoration groups (Kruskal–Wallis test, *p* = 0.806). With respect to site-related variables, restoration type was significantly associated with anterior/posterior implant location (*p* = 0.0004), whereas no significant association was observed for jaw location (maxilla vs. mandible; *p* = 0.0767).

**Figure 2:**
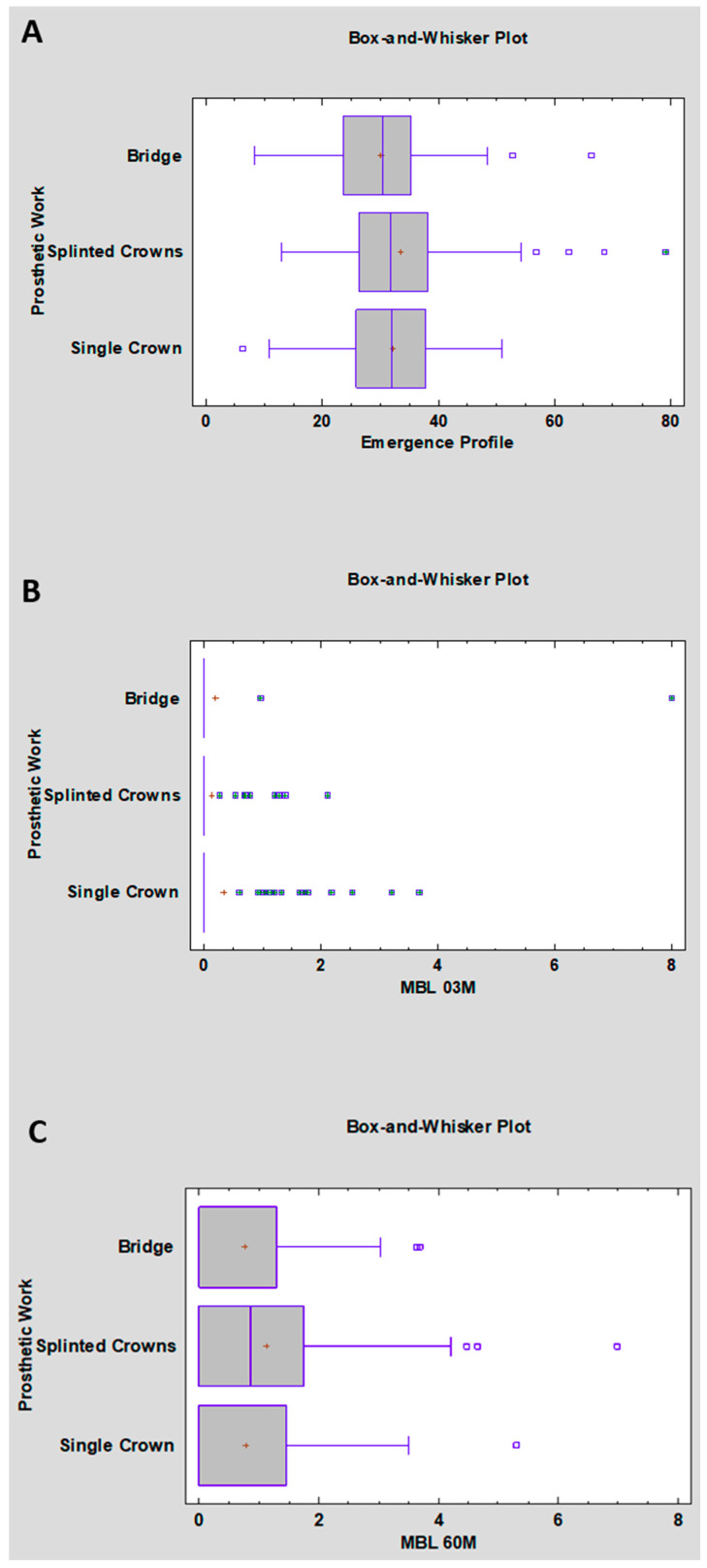
(**A**) Distribution of emergence angle values in the bridge, splinted crown, and single crown groups; (**B**) distribution of marginal bone loss at 3 months (MBL 03M) according to prosthetic restoration type; (**C**) distribution of marginal bone loss at 60 months (MBL 60M) according to prosthetic restoration type. Abbreviation: MBL-Marginal Bone Loss (mm); Emergence Angle in (°).

Table 3 and Figure 2A (Box-and-Whisker Plot) show the distribution of emergence angle values for the three types of prosthetic restorations. For bridges (*n* = 218), the mean emergence angle was 30 ± 11° (median: 30°); for splinted crowns (*n* = 208), 33 ± 11° (median: 32°); and for single crowns (*n* = 110), 32 ± 9° (median: 32°). Differences in means between groups were small (maximum ~3.3°). Standard deviations indicate moderate variability across all groups, with the greatest dispersion observed for splinted crowns.

Table 3 presents the descriptive distribution of all recorded mesial and distal emergence-angle measurements, whereas the inferential comparison shown in Table 4 was based on the averaged emergence-angle value calculated for each implant/restoration unit.

To verify whether the observed differences were statistically significant, a Kruskal–Wallis test was performed (Table 4). The obtained *p* value (0.202) far exceeds the conventional threshold for statistical significance (α = 0.05). The mean ranks for the groups were as follows: bridges—113.2, splinted crowns—134.9, and single crowns—130.3. Although the ranks differ, the test indicated that these differences are small enough to have arisen merely from random variation in the data. Therefore, no statistically significant differences were found in the median emergence angles among the three groups.

The analysis of marginal bone loss at 3 months (MBL 03M) is presented in Table 5 and Figure 2B. In all three groups, the median MBL was 0.0 mm, indicating that more than half of the patients in each group experienced no bone loss. Mean MBL values were low, ranging from 0.1 mm for splinted crowns to 0.3 mm for single crowns. The lowest variability was observed for splinted crowns (standard deviation: 0.4 mm), while the highest was seen in bridges (standard deviation: 1.2 mm), with individual cases reaching up to 8.0 mm.

ANOVA for MBL at 3 months (MBL 03M) yielded a *p* value of 0.195. Since this value exceeds the adopted significance level, there is no basis for rejecting the null hypothesis. Therefore, no statistically significant differences were found in mean bone loss at 3 months among the three types of prosthetic restorations.

After 5 years of follow-up (MBL 60M), a similar pattern was observed (Figure 2C). Visual inspection of the box plot does not reveal clear, systematic differences in the distribution of MBL values among the groups of bridges, splinted crowns, and single crowns. ANOVA yielded a *p* value of 0.081. It was therefore concluded that no statistically significant differences were found in mean bone loss after 5 years among bridges, splinted crowns, and single crowns. The observed variability is likely attributable to individual patient-related factors.

To comprehensively assess the effect of emergence angle on long-term bone loss, simple regression analyses were performed separately for each type of prosthetic restoration.

**Figure 3:**
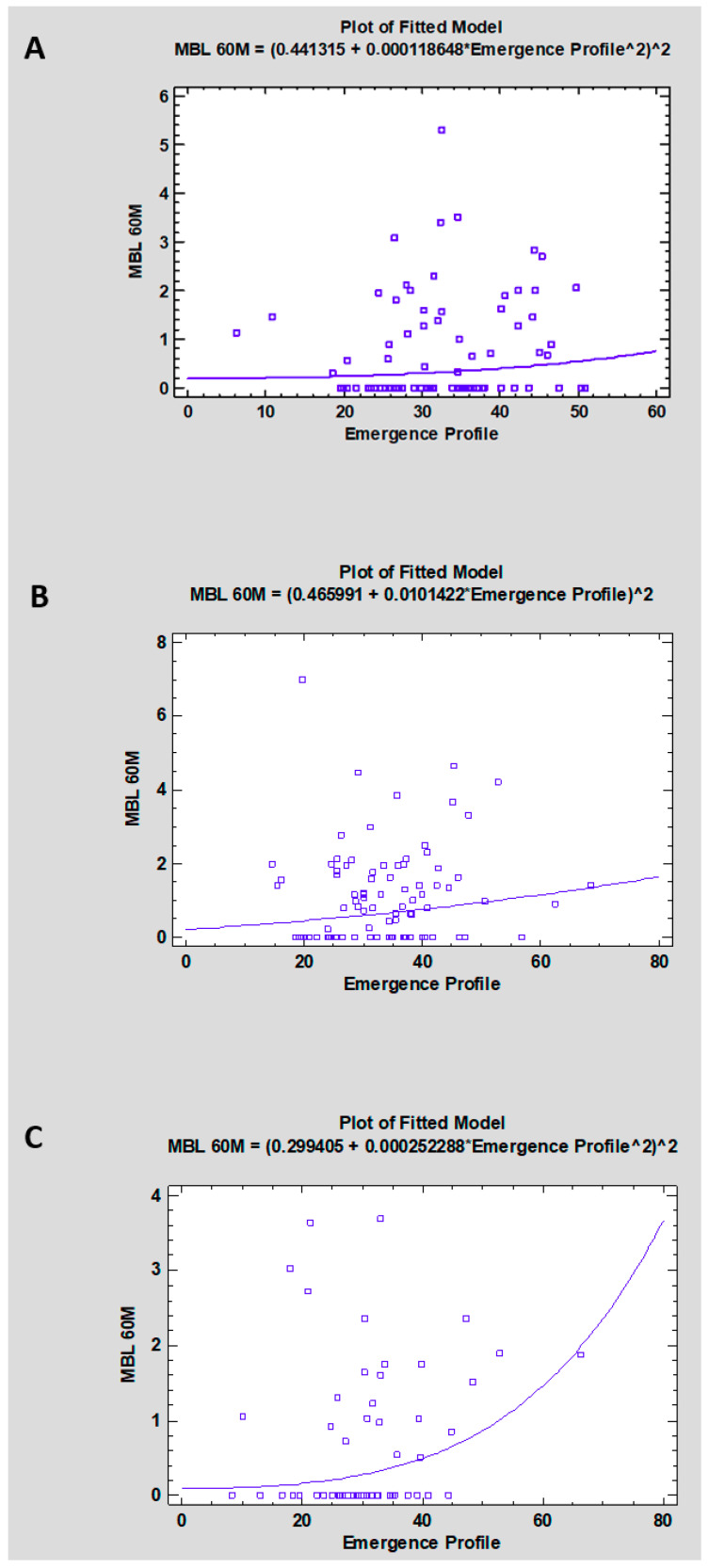
Exploratory regression plots illustrating the association between emergence angle and marginal bone loss (MBL) at 60 months in (**A**) single crowns, (**B**) splinted crowns, and (**C**) bridges. Abbreviations: MBL-Marginal Bone Loss (mm); Emergence Profile in (°).

Single crowns (Figure 3A): For single crowns, exploratory regression analysis did not demonstrate a statistically significant association between emergence angle and MBL at 60 months (*p* = 0.369; R^2^ = 1.1%), indicating negligible explanatory value.

Splinted crowns (Figure 3B): For splinted crowns, exploratory regression analysis did not demonstrate a statistically significant association between emergence angle and MBL at 60 months (*p* = 0.176; R^2^ = 2.1%). The model showed low explanatory value, indicating that emergence angle accounted for only a small proportion of the observed variability in MBL.

Bridges (Figure 3C): For bridges, exploratory regression analysis demonstrated a nominally statistically significant association between emergence angle and MBL at 60 months (*p* = 0.042; R^2^ = 7.9%). However, the explanatory value of the model was low, indicating that emergence angle accounted for only a small proportion of the observed variability in MBL. Therefore, this finding should be interpreted cautiously and regarded as clinically weak.

**Figure 4:**
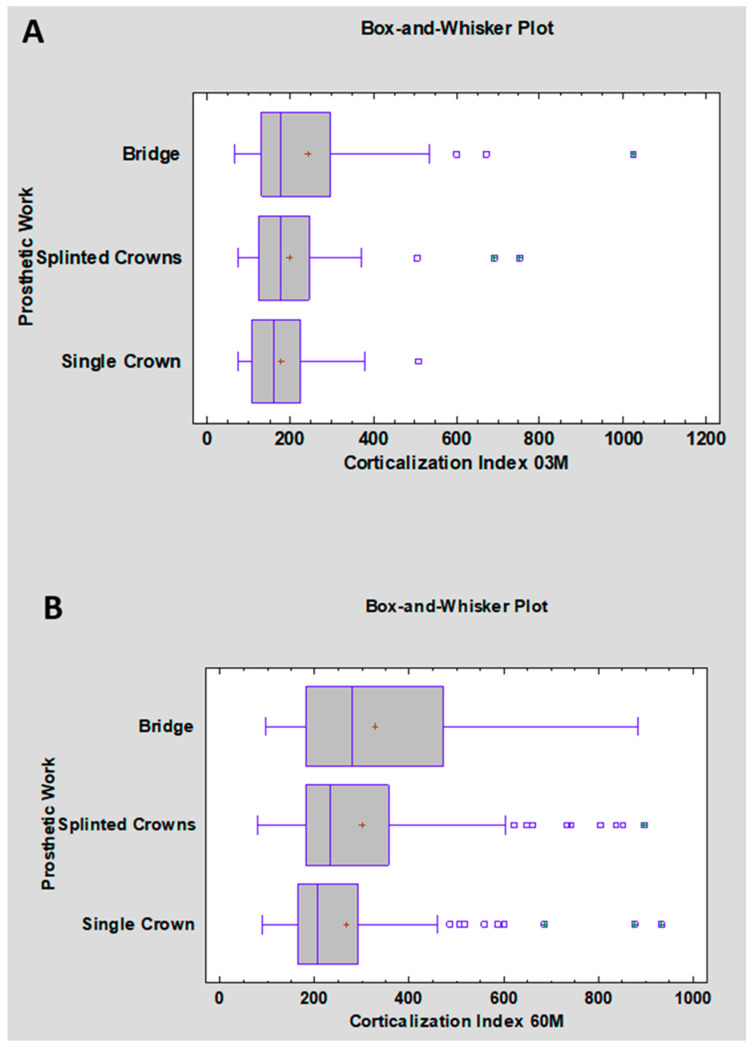
(**A**) Distribution of the Corticalization Index (CI) at 3 months according to prosthetic restoration type; (**B**) distribution of the Corticalization Index (CI) at 60 months according to prosthetic restoration type.

The analysis of the Corticalization Index after 3 months is presented in Table 6 and Figure 4A. The highest values for both the mean (243) and median (177) were observed for bridges, while the lowest were recorded for single crowns (mean: 178; median: 159). Bridges also showed the greatest data variability (standard deviation: 177). However, the Kruskal–Wallis test did not reveal statistically significant differences between the groups (*p* = 0.121).

After 60 months of observation (Table 6, Figure 4B), CI values increased in all groups, with bridges still showing the highest values (mean: 329.6; median: 281). The Kruskal–Wallis test revealed no statistically significant differences between groups (*p* = 0.072). These results indicate that the type of prosthetic restoration does not significantly influence the Corticalization Index, both at 3 months and over the long-term 5-year period.

An analysis of the relationship between the type of prosthetic restoration and selected clinical, including implant location (anterior or posterior region, maxillary or mandibular alveolar process) and habitual tobacco use, demonstrated statistically significant differences in some cases.

**Figure 5:**
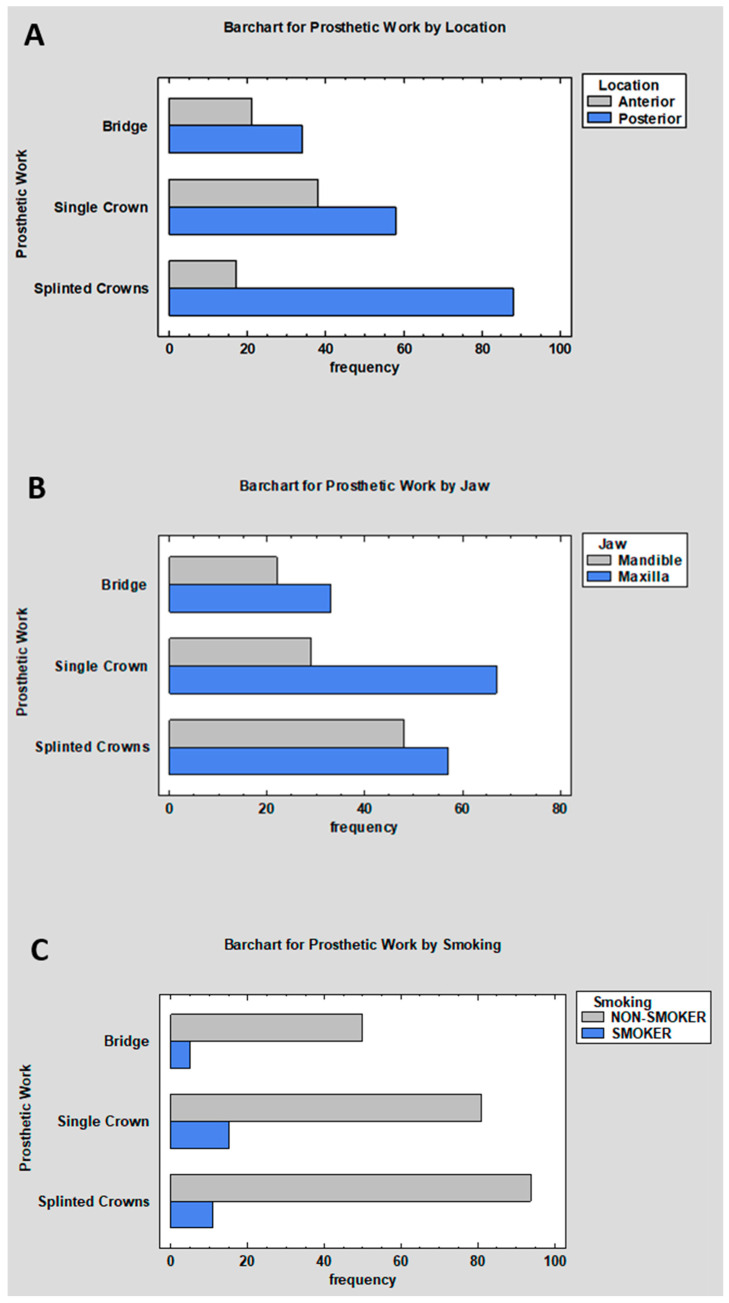
(**A**,**B**) Distribution of prosthetic restoration types according to implant location (anterior/posterior and maxilla/mandible); (**C**) distribution of prosthetic restoration types according to smoking status.

In the comparison between prosthetic restoration type and location within the dental arch (anterior vs. posterior region), the chi-square test demonstrated a statistically significant association (*p* = 0.0004). Both the tabulated data and their graphical representation indicate that single crowns predominated in the anterior region (14.84% of all cases), whereas splinted crowns were clearly more prevalent in the posterior region, accounting for 34.38% of all observations. Bridges were likewise more frequently found in the posterior region (Figure 5A,B; Table 7).

A different pattern was observed when the analysis was performed according to jaw location: maxilla and mandible. Here, the test of independence did not demonstrate a statistically significant association (*p* = 0.0767), indicating that the type of prosthetic restoration was not related to whether it was placed in the maxilla or the mandible. Although a higher overall proportion of cases was recorded in the maxilla (61.33%), these differences between restoration types were not large enough to be considered statistically significant (Table 7).

A descriptive association between smoking status and prosthetic restoration type was observed (*p* = 0.0409). However, because no formal multivariable adjustment was performed, this finding should be interpreted cautiously and should not be understood as indicating that smoking independently influenced the prosthetic design. As presented in Table 8, non-smokers predominated across all groups, comprising 87.89% of the study population. The highest proportion of smokers was found in the single-crown group (5.86%), whereas the lowest proportion was observed in the bridge group (1.95%) (Figure 5C, Table 8).

## 4. Discussion

The conducted retrospective study aimed to evaluate the influence of the prosthetic restoration emergence angle (EA) on marginal bone loss (MBL) and the Corticalization Index (CI) around dental implants over an observation period of up to 5 years.

The main conclusion of the present analysis, indicating a lack of statistically significant association between the emergence angle (EA) and marginal bone loss (MBL) for single crowns (*p* = 0.369) and splinted crowns (*p* = 0.176), is fully consistent with the findings reported by Lops et al. (2022) [13] in the publication “Marginal Bone Maintenance and Different Prosthetic Emergence Angles: A 3-Year Retrospective Study.” Strong corroboration of this observation comes from the extensive study by Kou et al. (2023) [10], “Prosthetic emergence angle in different implant sites and their correlation with marginal bone loss: A retrospective study,” in which a cohort of 502 single implant-supported crowns was analyzed. They also found no correlation between EA and MBL across anterior, premolar, and molar regions (all comparisons *p* > 0.05). In Lops’ study, including 312 implants with internal conical connections, no statistically significant difference in MBL was observed between the group with EA > 30° (mean 45°) and the group with EA ≤ 30° (mean 22°) after 3 years of follow-up (MBL change 0.06 ± 0.09 mm vs. 0.06 ± 0.10 mm; *p* = 0.969). These authors concluded that, in cases with a tight and stable implant–abutment connection, an emergence angle exceeding 30° does not negatively affect marginal bone stability [10,13].

However, a recent rigorous preclinical study by Lee et al. (2025) [14] provides new insights into this issue, offering direct histological evidence of the detrimental effects of a large emergence angle (EA, 60°) on both soft and hard tissues surrounding dental implants. The study was conducted on beagle dogs. Implants with crown emergence angles of 30° (NE) and 60° (WE) in splinted restorations were compared. Significantly greater bone remodelling was observed in the WE group, confirmed both radiographically and histomorphometrically. Importantly, the WE group also exhibited a shorter connective tissue attachment and a reduced fraction of collagen fibre area within the peri-implant epithelium. These findings indicate impaired formation of a tight soft tissue barrier (biologic width) around implants with a wide emergence angle, facilitating bacterial penetration and leading to secondary bone loss.

Soft tissue management techniques that preserve or augment keratinized mucosa have been shown to influence peri implant tissue stability and should be considered when interpreting radiographic bone changes [15].

The similarity of clinical findings across studies suggests that the influence of emergence angle on marginal bone behavior may be modulated by additional implant-related and clinical factors [10,13]. Both studies focused on implants with internal conical connections and platform switching, which—as demonstrated by other research—effectively minimize micromotion and microbiological leakage at the implant–abutment interface, potentially compensating for the adverse effects of a non-optimal crown profile. The study by Lee et al. [14], conducted in a controlled animal model without such confounding variables, clearly demonstrates the biological risk associated with a large EA, which in clinical practice may be masked by other favorable factors.

A key explanation for the lack of correlation observed in clinical studies, as highlighted by Kou et al. [10], is the anatomical variability of the emergence angle. They demonstrated that the mean EA in the molar region (34.53° ± 13.27) was significantly higher than in the anterior (21.67° ± 10.80) and premolar regions (26.29° ± 12.78). In molars, over 60% of implants had an EA > 30°, whereas in the anterior region, this proportion was approximately 20%. Physiologically, the greater width of molar teeth necessitates the use of wider implants, which often requires a larger emergence angle to restore the proper crown contour. The fact that no correlation with MBL was observed in the molar region despite the frequent occurrence of large EAs supports the interpretation that, under clinically stable conditions, emergence angle alone may not be a dominant determinant of marginal bone behavior. Nevertheless, the findings of Lee et al. [14] serve as an important caution, indicating that striving for extremely large angles (e.g., 60°), even in regions where anatomy permits it, may exceed the adaptive capacity of the surrounding tissues. It should also be noted that the range of emergence angle values observed in the present cohort was relatively narrow, and no predefined threshold-based categorical analysis was performed. Therefore, the present study was not designed to identify a clinically relevant emergence-angle cutoff above which peri-implant remodeling might increase.

In the present study, a gradual increase in the Corticalization Index (CI) over time was observed, with a median value rising from 165 at 3 months to 234 at 5 years. This process, reflecting the remodeling of trabecular bone toward a denser, cortical-like structure, was thoroughly characterized by Kozakiewicz et al. (2022) [7] in the publication “Measures of Corticalization.” Their research confirms that functional loading of implants induces changes in the alveolar bone structure, ultimately leading to its corticalization. At the same time, CI should be interpreted as a radiographic surrogate of bone adaptation rather than as a direct histological measure of bone quality or biological maturity. Therefore, radiographic corticalization cannot by itself determine whether the remodeled peri-implant bone represents a fully stable and histologically favorable structure.

A broader biological context is also provided by long-term histological and histomorphometric research on jawbone regeneration. Tumedei et al. emphasized that the long-term quality and stability of regenerated bone depend not only on radiographic appearance, but also on the biological environment, regenerative conditions, and tissue maturation over time. From this perspective, the increased corticalization observed in the present study may be interpreted as a radiographic sign of structural adaptation under functional loading, but it cannot by itself confirm that the remodeled peri-implant bone has achieved the same histological quality, maturity, or long-term stability as physiologically well-organized bone. Therefore, radiographic corticalization should be interpreted cautiously and in conjunction with the broader biological and clinical context [16].

The most recent study by Wach et al. (2024) [8], “New Radiological Corticalization Index as an Indicator of Implant Success Rate Depending on Prosthetic Restoration—5 Years of Follow-Up”, provides key context for interpreting our findings regarding the variable impact of prosthetic restoration type. Analyzing more than 2000 samples, the authors demonstrated that corticalization is a heterogeneous phenomenon, with its relationship to marginal bone loss being strongly dependent on the type of prosthetic reconstruction.

In the case of single crowns, both in our study and in the work of Wach et al. [8], an increase in the Corticalization Index (CI) over time was observed. Importantly, Wach et al. reported that the increase in CI in this group was not accompanied by significant marginal bone loss (MBL) over a 5-year follow-up. This supports our conclusion that, for single crowns, the emergence angle alone is not a key risk factor, which is also reflected in the findings of Kou et al. [10] for single crowns across all anatomical locations.

In the group of splinted crowns in our study, no significant relationship between emergence angle (EA) and marginal bone loss (MBL) was observed. Interestingly, Wach et al. [8] also did not detect an effect of this type of restoration on the Corticalization Index (CI), despite the presence of associations in MBL. The study by Lee et al. [14] provides a key distinction: while splinting alone was not the main differentiating factor, when combined with a wide EA (WE), the greatest bone remodeling was observed precisely in the splinted regions (T-splint and M-splint). The authors suggest that the distribution of occlusal forces in splinted restorations may interact differently with bone, making them a favorable prosthetic solution while simultaneously creating an environment in which the negative effect of a suboptimal EA may be amplified.

Prosthetic bridges represent a particularly interesting group. In our study, we observed a weak but statistically significant association (*p* = 0.042) between emergence angle (EA) and marginal bone loss (MBL) for bridges, although it accounted for only 7.9% of the variance. Importantly, the subgroup-specific regression analyses should be interpreted with caution. Although a nominally significant association was observed for bridges, the low coefficient of determination indicates limited explanatory value, suggesting that emergence angle alone accounted for only a small fraction of the observed variability in long-term MBL. This supports an interpretation in which the detected association is weak and potentially sensitive to other unmeasured biological or biomechanical factors. The findings of Wach et al. [8] provide a possible explanation for this observation. In their study, bridges were associated with an increased Corticalization Index (CI) and greater MBL compared to other restorations. Furthermore, they noted that six-unit bridges exerted the most unfavorable effect on MBL. In the context of Kou et al. [10], which did not include bridges, and Lee et al. [14], which focused on splinted implants, our observation suggests that in extensive, multi-unit prosthetic restorations—where biomechanical forces are substantially more complex—prosthetic factors, including potentially EA, may begin to play a more significant role. In contrast, for single crowns, their influence is largely overshadowed by other, more dominant factors. The findings of Lee et al. [14] for splinted crowns further confirm that in such complex configurations, biomechanics are more intricate, and access for hygiene becomes limited.

An interesting aspect of the study by Wach et al. [8] is the observation that corticalization per se does not necessarily have a negative impact. For instance, in cases using platform switching (PS) designs, despite an increase in the Corticalization Index (CI), a lower marginal bone loss (MBL) was observed (though not statistically significant). Similarly, cemented crowns were associated with higher CI values after 5 years compared to screw-retained crowns, without a significant difference in MBL. The authors hypothesize that PS may mitigate the potentially adverse effects of progressive corticalization over time, and that an increase in CI under certain conditions may reflect adaptive, rather than pathological, bone remodelling.

These observations provide an excellent explanation for why, in our study, despite the lack of correlation between the emergence angle (EA) and marginal bone loss (MBL) in most groups, an independent corticalization process was observed. They indicate that these two phenomena—bone loss and changes in bone structure—may be driven by partially distinct factors. The study by Kou et al. [10] adds another dimension: the anatomical factor. The crown shape may not have a decisive influence on local marginal bone height loss, whereas long-term functional loading, the type of prosthetic restoration, and the physiologically determined implant location may represent important contributors to both internal bone architecture remodelling (CI) and marginal bone loss (MBL). The study by Lee et al. [14] precisely identifies one mechanism through which a large EA can disrupt this balance—namely, by impairing the connective tissue attachment and the integrity of the epithelial barrier—thereby facilitating inflammation and pathological bone remodelling.

Across all analyzed studies, it should be emphasized that prosthetic factors represent only one of many elements influencing treatment outcomes. Implant treatment success is equally, if not more, affected by primary implant stability, bone quality and density, the patient’s systemic health, habits such as smoking, and oral hygiene levels [17,18]. Moreover, as demonstrated by Kou et al. [10], the implant site itself (anterior vs. posterior) dictates the physiologically feasible range of emergence angles, which should be considered during treatment planning. The study by Larsson et al. (2023) [19] identified additional key risk factors: probable bruxism and male sex were significantly associated with increased risk of failure for single crowns on implants. The authors also observed that bruxism was linked to a higher frequency of ceramic fractures, which, alongside loosening, unscrewing, or decementation of the crown, constitutes the most common technical complication. The radiomics and texture analysis applied by Kozakiewicz and Wach provide new opportunities for an objective assessment of bone condition beyond simple linear MBL measurements. Indices such as the Corticalization Index (CI) enable detection of subtle structural changes in bone that may precede radiologically visible defects [7,8].

The main limitation of this study, similar to the research by Lops and Kou et al. [10,12], is the reliance on two-dimensional radiographs. Such assessments do not allow evaluation of the bone condition on the vestibular and palatal/lingual aspects, which are critical for tissue health and aesthetics. It should be noted that even the use of three-dimensional cone-beam computed tomography (CBCT), which theoretically allows such assessment, faces significant limitations due to artifacts caused by the presence of implants. As highlighted in the literature review by Sawicki et al. (2022) [20], metallic implants produce characteristic CBCT artifacts in the form of streaks, shadows, and noise, which considerably degrade image quality and hinder accurate evaluation of the bone adjacent to the implant. The severity of these artifacts depends on exposure parameters (voltage, current), implant material, and its orientation relative to the radiation beam, posing an additional methodological challenge. The preclinical study by Lee et al. [14] provides valuable histological insights; however, its applicability to clinical conditions in humans is limited, where factors such as oral hygiene, implant type, and long-term functional loading play a crucial role. Three-dimensional imaging has also been used in other maxillofacial applications to characterize treatment-related bone changes more comprehensively; however, in the peri-implant setting its interpretive value remains constrained by implant-related artifacts and by the specific requirements of interproximal bone assessment [21].

All of the retrospective studies discussed so far, including the present one, relied on two-dimensional measurements of the emergence angle (EA) from radiographs, which constitutes a key limitation. The breakthrough study by Mancini et al. (2023) [22], “The 3D emergence profile on implant-supported restorations: A method for evaluating restorative angles,” provides a novel and highly precise tool for three-dimensional assessment of the emergence profile. Their method, based on analysis of digital 3D (STL) models of restorations, enabled for the first time measurement of the EA not only in interproximal areas (mesial and distal) but also on the labial and buccal aspects. The authors demonstrated that emergence angles on the vestibular side are significantly smaller (steeper) than those in interproximal spaces, particularly in the anterior region. This indicates that traditional 2D radiograph-based assessments of EA, which focused primarily on interproximal surfaces, may have failed to capture the steepness of the profile in areas critical for aesthetics and soft tissue health.

In the context of our discussion, the application of such an advanced 3D method in future studies could provide definitive answers regarding the actual influence of crown morphology on peri-implant tissues. It is possible that not the averaged EA, but rather a specific excessively steep contour on the labial side, represents the key risk factor for gingival recession and inflammation, as suggested in other studies [23,24]. Mancini et al.’s method [22] offers a tool to test this hypothesis. Furthermore, their concept of separately analyzing the biological zone (closer to the implant platform) and the aesthetic zone (closer to the gingival margin) allows for high precision in the design and evaluation of restorations, enabling optimization of crown morphology for both tissue health and aesthetics.

The present findings suggest that the choice of implant-supported prosthetic restoration was more closely related to anterior–posterior position within the dental arch than to placement in the maxilla or mandible. The predominance of single crowns in the anterior region, together with the more frequent use of splinted crowns and bridges in the posterior region, appears consistent with clinical decision-making in which esthetic demands are prioritized anteriorly, whereas posterior reconstructions are more often planned with greater consideration of load distribution. By contrast, the absence of a statistically significant association between restoration type and jaw location suggests that allocation to the maxilla or mandible, in itself, may be of lesser importance than local clinical conditions when determining the prosthetic design. The association observed with smoking status should also be interpreted cautiously. Although statistically significant, it should not be understood as indicating that smoking directly determines the type of prosthetic restoration selected. Rather, this finding may reflect the broader clinical relevance of smoking as a biological risk factor in implant therapy, given its established association with less favorable healing and peri-implant bone outcomes. Overall, these results support the view that restoration type is influenced primarily by site-specific functional and clinical considerations, while smoking should be regarded as an accompanying risk-related factor rather than a direct determinant of prosthetic design [25,26,27,28,29].

This study has several limitations. First, its retrospective design precluded a formal a priori sample size calculation and a prospectively registered statistical analysis plan; therefore, the findings should be interpreted as exploratory and hypothesis-generating, particularly with respect to subgroup-based regression analyses. Second, the study was based on two-dimensional radiographs, which do not allow assessment of the buccal and lingual bone plates and therefore provide only a partial representation of peri-implant bone remodeling. Third, although the radiographic measurements were performed by two examiners with separate measurement responsibilities (emergence angle vs. MBL/CI) and repeated measurements showed approximately 90% repeatability, no formal inter-observer reliability analysis using agreement coefficients was performed. Fourth, mesial and distal radiographic measurements were initially recorded separately, but the principal analyses relied on their arithmetic mean at the implant/restoration level. As a result, potential side-specific differences in peri-implant bone behavior were not evaluated separately. In addition, because multiple raw measurements could originate from the same implant and/or patient, and the inferential analyses did not explicitly account for intra-patient clustering, the reported *p*-values should be interpreted with caution. Fifth, the study did not include formal multivariable adjustment for potential confounders such as implant geometry, insertion torque, and site-related variables. In addition, prosthetic connection characteristics, including platform-switching configuration and related abutment variables, were not consistently available in the retrospective records and could therefore not be evaluated as potential modifiers of emergence configuration or marginal bone behavior. Moreover, soft-tissue phenotype/periodontal biotype was not consistently documented in the retrospective records and could not be incorporated into the present analysis, despite its potential relevance for peri-implant tissue stability and bone remodeling. Although several of these variables were available descriptively, the present analysis was not designed to determine whether emergence angle remained an independent predictor after adjustment. Therefore, the findings should be interpreted as exploratory rather than fully adjusted estimates of effect. Finally, the regression analyses were based on exploratory transformation-based models selected to accommodate non-normal outcome distributions. These models should be regarded as descriptive rather than confirmatory, and their low explanatory power limits the strength of causal or predictive interpretation. In addition, the exclusion of patients with major systemic conditions affecting bone metabolism and diagnosed bruxism means that the present cohort reflects a relatively controlled clinical scenario; therefore, the findings may not be directly generalizable to higher-risk implant populations. Future studies using prospectively structured datasets and validated three-dimensional assessment methods, such as STL-based restorative angle analysis and carefully selected CBCT protocols, may provide a more comprehensive evaluation of peri-implant tissue changes, including esthetic and soft tissue-related aspects.

## 5. Conclusions

This retrospective study analyzed 155 patients and radiographic data derived from mesial and distal measurements collected over up to 5 years of follow-up. The emergence angle was similar across the evaluated prosthetic groups, and no significant differences in marginal bone loss (MBL) or Corticalization Index (CI) were observed between restoration types at either 3 months or 5 years. No statistically significant association between emergence angle and MBL was observed for single crowns or splinted crowns. For bridges, the association reached statistical significance, but its explanatory value was low and its clinical relevance appears limited. CI increased over time in all groups, which may be consistent with ongoing radiographic bone adaptation under functional loading. Most variability in MBL and CI was not explained by the prosthetic variable analyzed in this study, suggesting that other patient-related, biological, and biomechanical factors may also contribute. Within the limitations of this retrospective radiographic analysis, no clinically meaningful association was observed between emergence angle and marginal bone loss for single or splinted crowns. The association observed for bridges was statistically significant but weak and should be interpreted cautiously.

## Figures and Tables

**Table 1:** Basic statistics for the entire cohort: emergence angle, marginal bone loss (MBL), and Corticalization Index (CI) over time. Abbreviations: M, months.

	Emergence Angle (°)	MBL 00M (mm)	MBL 03M (mm)	MBL 60M (mm)	Corticalization Index 03M	Corticalization Index 60M
Average	31.80	0.0	0.2	0.9	201	295
Standard deviation	10.4	0.0	0.7	1.2	126	180

**Table 2:** Characteristics of the implants used—diameter, length, and insertion torque.

	Diameter (mm)	Length (mm)	Torque (Ncm)
Count	263	262	230
Average	3.758	12.429	40.478
Standard deviation	0.189	2.401	11

**Table 3:** Distribution of recorded mesial and distal emergence-angle measurements according to prosthetic restoration type.

Prosthetic Work	Count	Average	Standard Deviation
Bridge	218	30.1	10.7
Splinted Crowns	208	33.5	10.8
Single Crown	110	32.04	8.9
Total	536	32.2	10.2

**Table 4:** Results of the Kruskal–Wallis test for emergence angle according to prosthetic restoration type, based on analyzed prosthetic restorations.

Prosthetic Work	Number of Analyzed Restorations	Average Rank
Bridge	55	113.1
Splinted Crowns	105	134.9
Single Crown	96	130.3

**Table 5:** Marginal bone loss at 3 months according to prosthetic restoration type. *p* value from one-way ANOVA.

Prosthetic Work	Count	Average 03M	Standard Deviation	*p* Value
Bridge	44	0.2	1.2	0.195
Splinted Crowns	94	0.1	0.4	
Single Crown	88	0.3	0.7	
Total	226	0.2	0.7	

**Table 6:** Descriptive statistics of the Corticalization Index (CI) at 3 and 60 months, stratified by prosthetic restoration type.

Prosthetic Work	Count	Average 03M	Standard Deviation 03M	Average 60M	Standard Deviation 60M
Bridge	218	242.3	176.9	329.6	176.0
Splinted Crowns	208	198.5	114.8	301.2	189.7
Single Crown	110	178.0	88.9	266.6	168.6

**Table 7:** Frequency distribution of prosthetic restoration types according to location. *p* values from chi-square tests.

	Anterior	Posterior	Row Total	Mandible	Maxilla	Row Total
Bridge	21	34	55	22	33	55
	8.20%	13.28%	21.48%	8.59%	12.89%	21.48%
Single Crown	38	58	96	29	67	96
	14.84%	22.66%	37.50%	11.33%	26.17%	37.50%
Splinted Crowns	17	88	105	48	57	105
	6.64%	34.38%	41.02%	18.75%	22.27%	41.02%
*p* value	0.0004			0.0767		

**Table 8:** Distribution of prosthetic restoration types according to smoking status. *p* value from chi-square test.

	Non-Smoker	Smoker	Row Total
Bridge	50	5	55
	19.53%	1.95%	21.48%
Single Crown	81	15	96
	31.64%	5.86%	37.50%
Splinted Crowns	94	11	105
	36.72%	4.30%	41.02%
*p* value	0.0409		

## Data Availability

The study was based on retrospective clinical and radiographic documentation. Individual-level raw data are not publicly available due to ethical and institutional restrictions related to the source records. To improve transparency, aggregated summary data are provided in the manuscript and Supplementary Materials. Further inquiries may be directed to the corresponding author.

## Supplementary Materials

The following supporting information can be downloaded at: https://www.mdpi.com/article/10.3390/jcm15103764/s1, Supplementary Table S1. Reported descriptive statistics for the principal radiographic variables according to prosthetic restoration type.
