# Supplementary material for: Emergence Angle, Marginal Bone Loss, and Radiographic Corticalization Around MIS Implants: A 5-Year Retrospective Study of Single, Splinted, and Bridge Restorations
Source: J Clin Med. 2026 May 14;15(10):3764. doi: 10.3390/jcm15103764 (PMC13207012; doi:10.3390/jcm15103764)
Supplement: Supplementary file 1 [file jcm-15-03764-s001.zip › jcm-4258593-supplementary.pdf]

Supplementary Table S1. The table includes only values explicitly reported in the manuscript and associated statistical outputs, without additional calculations from the raw dataset. Descriptive parameters are presented for emergence angle, marginal bone loss at 3 months (MBL 03M), and the Corticalization Index at 3 and 60 months (CI 03M, CI 60M), stratified by prosthetic restoration type.

Prosthetic restoration	Emergence angle Count	Emergence angle Mean ± SD (°)	MBL 03M Count	MBL 03M Mean ± SD (mm)	CI 03M Count	CI 03M Mean ± SD	CI 60M Count	CI 60M Mean ± SD
Bridge	218	30.1 ± 10.7	44	0.2 ± 1.2	218	242.3 ± 176.9	218	329.6 ± 176.0
Splinted crowns	208	33.5 ± 10.8	94	0.1 ± 0.4	208	198.5 ± 114.8	208	301.2 ± 189.7
Single crown	110	32.04 ± 8.9	88	0.3 ± 0.7	110	178.0 ± 88.9	110	266.6 ± 168.6
